# Direct medical cost of COVID-19 in children hospitalized at a tertiary referral healthcare center in Mexico City

**DOI:** 10.3389/fpubh.2023.1117906

**Published:** 2023-08-17

**Authors:** Alfonso Reyes-López, Rodolfo Norberto Jimenez-Juárez, Guillermo Salinas-Escudero, Martha Josefina Avilés-Robles, Silvia Martínez-Valverde, Víctor Granados-García, Juan Garduño-Espinosa

**Affiliations:** ^1^Center for Economic and Social Studies in Health, Hospital Infantil de México Federico Gómez, Mexico City, Mexico; ^2^Department of Infectious Diseases, Hospital Infantil de México Federico Gómez, Mexico City, Mexico; ^3^Epidemiological and Health Services Research Unit Aging Area, Centro Médico Nacional Siglo XXI, Instituto Mexicano del Seguro Social, Mexico City, Mexico; ^4^Division of Research, Hospital Infantil de México Federico Gómez, Mexico City, Mexico

**Keywords:** medical, cost, COVID-19, Mexican, children

## Abstract

**Introduction:**

Despite the end of the COVID-19 pandemic being declared by the WHO, the economic consequences are far from over. One of these implications was the cost of inpatient care for health institutions. To date, some studies have examined the economic burden of COVID-19 in the adult population but only a few have focused on child populations.

**Objective:**

To estimate the direct medical costs of COVID-19, focusing on children in Mexico.

**Method:**

Data about resources consumed during hospital stays were extracted from the medical records of patients hospitalized at a Mexican tertiary healthcare institution. Other sources of information were the unit prices of inputs and the salaries of health personnel. A micro-costing methodology was used to obtain cost results by age group over different hospital areas. Data analysis was performed with descriptive statistics and regression models to evaluate the predictors of total cost.

**Results:**

One hundred and ten medical records were reviewed of which 57.3% corresponded to male patients and the mean age was 7.2 years old. The estimated average cost per patient was US$5,943 (95% CI: US$4,249–7,637). When the costs of the three clinical areas were summed, only the 5–10 years old group showed a maximum cost of US$14,000. The regression analysis revealed the following factors as significant: sex, age, staying at an emergency room, having a positive bacterial culture, and having comorbidities.

**Discussion:**

The cost results were somewhat similar to those reported in children from the USA, but only regarding low severity COVID-19 cases. However, comparability between these types of studies should be done with caution due to the huge differences between the healthcare systems of countries. The study cost results may help public decision-makers in budget planning and as inputs for future cost-effectiveness studies about interventions regarding COVID-19.

## Introduction

Although the World Health Organization has declared the end of the COVID-19 pandemic as a “global health emergency,” (1) the economic issues around it are still a matter of concern and analysis, especially in the noticeable constraints to economic activities during the pandemic and the expected consequences post-covid (2–4)**.** The healthcare costs related to COVID-19 are a major problem faced by health authorities at the national level, and since the adult population is the most affected, more studies about the economic burden of COVID-19 have been published in this population compared with children. Here, we included some relevant cost estimations for adults. For example, in China, a considerable economic impact has been found during the first quarter of 2020, where the cost results of confirmed cases were US$939.06 for a non-severe case, US$8,878.66 for a severe case, and US$25,578 for a critical case (5)**.** A French study reported cost per patient according to three levels of care: no or low-flow oxygen support (level 1: €3,989), non-invasive ventilation (level 2: €10,577), and mechanical ventilation (level 3: €21,133) (6)**.** Another European study reported similar cost results although using different severity criteria: general hospitalization was €4,122.96 while intensive care unit (ICU) admission was €21,199.20 (7)**.** In Latin America a Brazilian study reported a mean cost of US$7,394 for general hospitalization and US$12,179 for ICU admission (8)**.**

The child population contains fewer cost studies with most being from the United States of America (USA) and only one from Korea. The USA studies used specific approaches in highlighting different features related to COVID-19 like the number of organ systems affected (OSA), the use of invasive mechanical ventilation (IMV), children with chronic conditions or the role of obesity, while the Korean study reported cost results by age groups. The inpatient cost of COVID-19 in children with less than two OSA was US$6,474 but in those with 6–8 OSA the median cost was US$98,643 (9)**;** a median cost of US$4,913 was estimated for an inpatient admission without ICU or IMV, US$14,682 with ICU but without IMV, US$9,555 without ICU but with IMV, and US$43,279 with ICU and IMV (10)**.** The mean cost of COVID-19 in children with complex chronic conditions was US$17,009 (99% CI:US$14,436 – 20,041) (11) and children with severe obesity had a 53% or US$7,660 (95% CI:US$1,721–US$13,600) higher cost of care compared with those with healthy weight (12)**.** Surprisingly the cost results from the Korean study were well below the results obtained in the studies from the USA, with estimates by the age groups 0–5 years old US$749, 6–10 US$2,584, 11–15 US$2,050, and 16–19 US$3,655 (13)**.**

To date, no such studies have been carried out in Mexico. The Mexican healthcare system is highly segmented; wealthier, formal private sector workers receive care from the Mexican Institute for Social Security (IMSS) and federal public workers receive it from the Institute for Social Security and Services for Civil Servants (ISSSTE), whereas the non-salaried population (self-employed, underemployed, unemployed, and those out of the labor market permanently or temporarily) typically have access to health services through the Ministry of Health on a public assistance basis. Healthcare for this population was funded from 2006 to 2019 by the Popular Health Insurance, but it was canceled by the federal government, which caused shortages of medicines and families having to pay out-of-pocket. The study hospital belongs to the Ministry of Health subsystem, and a lack of funding has been a factor during the pandemic and thereafter (14–18)**.**

Hence, a concise picture of the cost of COVID-19 will help decision makers to negotiate an optimal allocation of resources for a disease that has been added to existing ones. Therefore, this study aims to estimate the direct medical costs of COVID-19 in children treated in a tertiary referral hospital in Mexico.

## Materials and methods

This was an observational, retrospective, and longitudinal study, so the data were extracted from the medical records of children hospitalized for COVID-19 at Mexico’s Children’s Hospital Federico Gómez, which is a tertiary referral hospital at the national level. The hospital has 20 beds in the emergency room, 20 beds in the Pediatric Intensive Care Unit (PICU), and 30 beds for neonates, of which, 12 correspond to the Neonatal Intensive Care Unit (NICU). An additional ward with 18 beds was required for COVID-19 patients not requiring intensive care.

**Figure 1:**
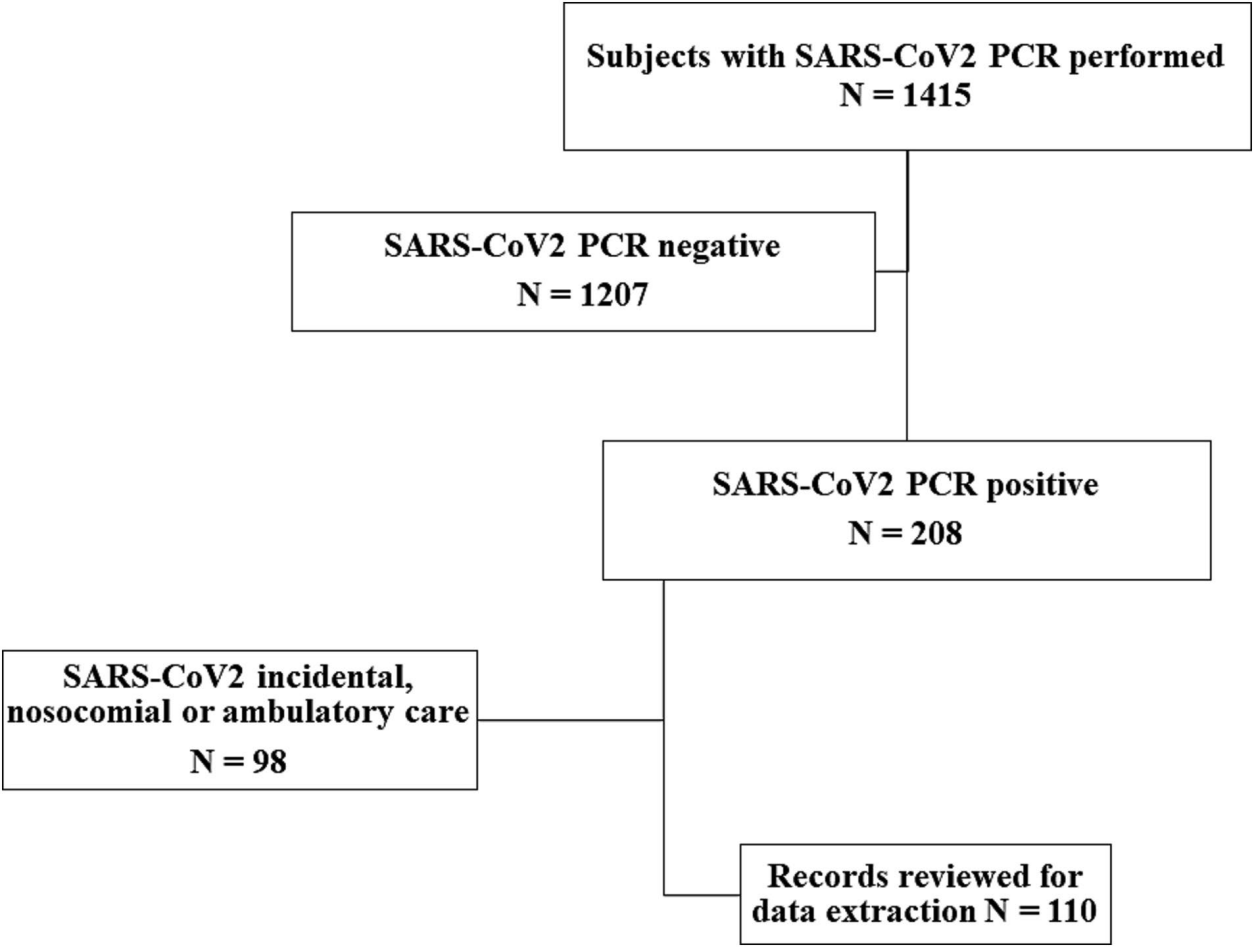
Flow chart showing the steps to draw the sample of medical records reviewed for data extraction.

The only inclusion criterion was medical records belonging to patients with a positive PCR test for SARS-CoV2, and criteria for not being included were incomplete data about the use of medical resources, having received outpatient care, and patients with hospital-acquired SARS-CoV2 infections. A flow chart describing the initial and final sample of medical records included for analysis is shown in Figure 1. Medical records reviews were included from March to August 2020 and the medical protocol used for delivering care to patients was as follows: a PCR test was carried out only for patients with respiratory o digestive symptoms suggesting COVID-19, but from June all patients were screened with PCR tests. Medical procedures for COVID-19 patients included lab tests and chest X-rays only for those with pneumonia, providing supportive care to all, and prescribing antibiotics only to patients with comorbidities. Some child patients with multisystem inflammatory syndrome received gamma-globulin.

One nurse and one pediatrician were hired and trained for the review of medical records and data extraction, which were captured in a spreadsheet previously designed to facilitate information management. A person-based (‘bottom-up’) method was adopted, which consists of assigning resource use to individuals with the health condition of interest, but also the method enables the capturing of the resource intensity within each service encounter when data is available at an individual level, including to detect variability related to differences in important demographic characteristics between patients (19)**.** Therefore data collection regarding hospital stay covered all the areas where patients received care such as the emergency room (ER), PICU/NICU, and others. Likewise, the demographic and clinical data of patients were analyzed. The study focused mainly on the resources consumed only during hospitalization, and patients were not followed up on after discharge. The pandemic disrupted the hospital’s normal daily activities and delivery of care to COVID-19 patients, which required many routine procedures to be modified. Interviews with those in charge of the relevant medical departments were conducted to understand the extent to which these modifications affected healthcare costs and estimate the mean working hours per day of employees assigned to the hospital’s COVID-19 areas. The interviews asked them about the specific activities developed by health personnel and the average time consumed by undertaking them, alongside the use of personal protective equipment (PPE). The cost per man-hour for doctors and nurses and the daily per-capita cost of PPE were then calculated using information about the monthly gross salaries paid by the hospital and the cost of supplies purchasing, respectively. These costs were then added to the bed-day cost. The financial department of the hospital was the source of information.

The study used the micro-costing approach for which a generic cost function was first defined as: C = {(x_1_ + p_1_) + (x_2_ + p_2_) + ⋯ + (x_i_ + p_i_)}, where C is any of the cost components of healthcare, x_i_ is the number of resources consumed and p_i_ is the respective unit price (20)**.** The cost components were then aggregated over healthcare encounters (ER, PICU/NICU, general ward) to understand the total cost of care. All economic results were expressed as 2020 US dollars using an exchange rate of 22.61 peso/dollar.

Since the statistic of interest for decision-makers is the arithmetic mean, we used the nonparametric bootstrap procedure to make statistical inferences around the mean costs, which offers the advantage of avoiding parametric assumptions about the distribution of costs, while giving the possibility of making inferences about the arithmetic mean cost (21). This procedure is recommended either to evaluate the robustness of standard parametric tests or as a primary statistical test to make inferences about the arithmetic means of costs when the sample size is small to moderate (21). Comparisons between age groups were undertaken using the Kruskal-Wallis test. To evaluate the effect of patient characteristics on the total cost of COVID-19, a generalized linear model (GLM) was used, which also dealt with the problem of biased data and directly models heteroscedasticity (22). Six different GLMs were adjusted using the logarithmic link and square root functions with the Gaussian, Poisson, and gamma distribution families, but the models with the lowest Akaike and Bayesian information criteria (AIC, BIC) were the log-gamma and square root gamma, therefore the log-gamma model was used. All procedures were performed with Stata software 17.

## Results

**Figure 2:**
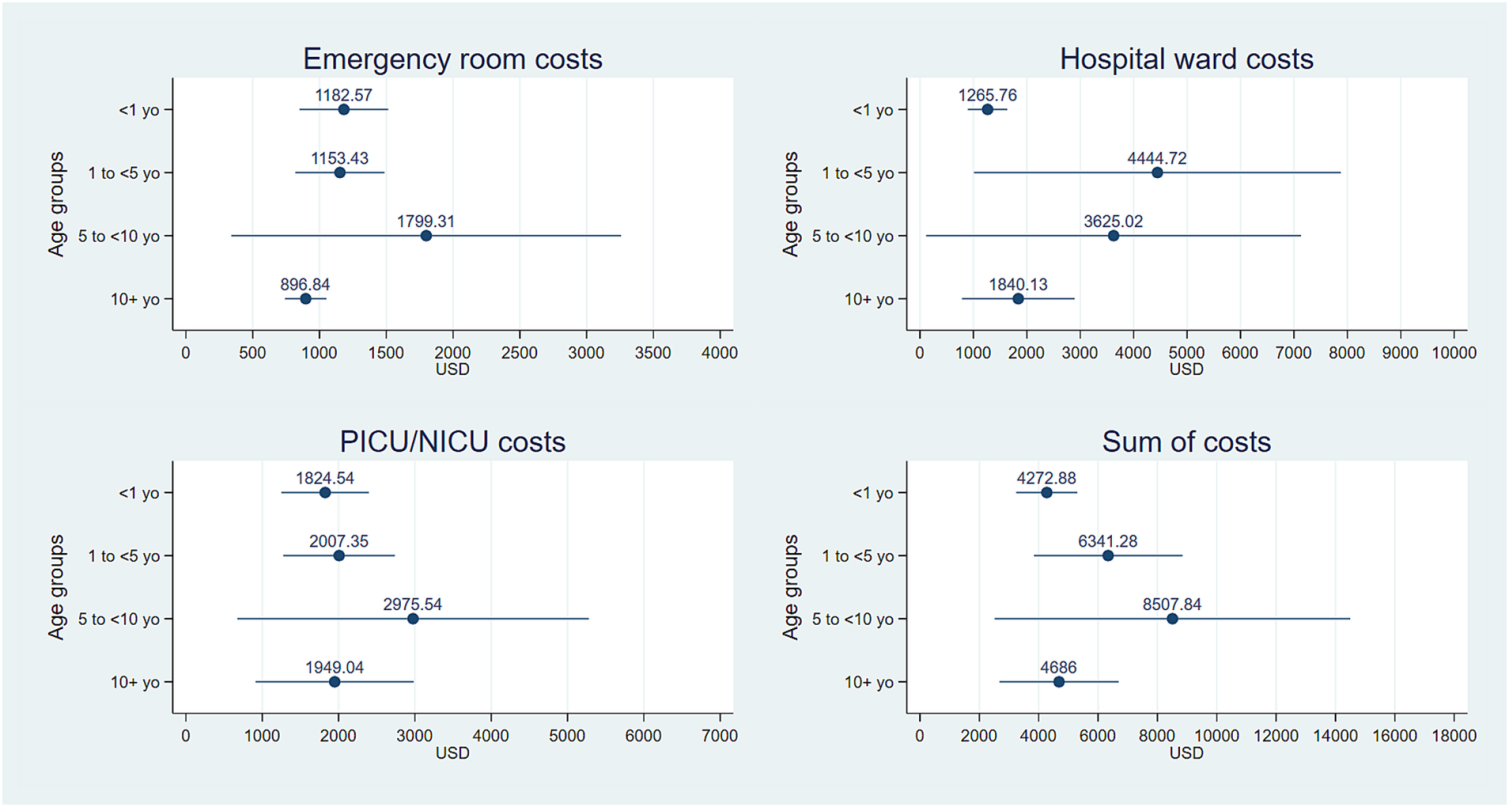
Mean total costs for each clinical area and the sum of costs over the three areas by age group. Horizontal lines represent 95% confidence intervals around the arithmetic mean obtained from the nonparametric bootstrap procedure with 1,000 iterations.

In total, 110 medical records on COVID-19 patients were reviewed for data extraction. The distribution by sex showed a higher proportion of male patients (57.3%) and the mean age was 7.2 years old (standard deviation: 5.7). Additional clinical characteristics of patients were summarized by age group in Table 1. The components of medical cost by age group and for each clinical area were detailed in Table 2. A mean total cost over the three clinical areas and all age groups of $5,943 (95% CI: $4,249 - $7,637) was estimated while the mean total cost for each clinical area is shown in Figure 2 including the sum of cost over by age groups, where the horizontal lines represent the 95% confidence intervals obtained from the nonparametric bootstrap procedure with 1,000 iterations. The 5 to 10 years old group exhibited the greatest uncertainty.

**Table 1:** Characteristics of patients included in the medical records review.

Variables	<1 yo (*n* = 20)	1 to < 5 yo (*n* = 28)	5 to < 10 yo (*n* = 27)	10+ yo (*n* = 35)	*p* value
Female, *n* (%)	15 (75)	5 (17.8)	13 (48.1)	14 (40)	<0.001
Male, *n* (%)	5 (25)	23 (82.1)	14 (51.8)	21 (60)	
Length of stay (days), mean (sd)	21.8 (26.3)	13.3 (15.8)	15.6 (19.6)	14.3 (32.9)	0.41
PIMS CDC, *n* (%)	0 (0)	7 (25)	4 (14.8)	15 (42.8)	<0.001
Comorbidities, *n* (%)	11 (55)	20 (71.4)	24 (88.9)	27 (77.1)	0.07
Positive bacteria culture test, *n* (%)	5 (25)	17 (60.7)	18 (66.7)	22 (62.8)	0.021
Intensive care, *n* (%)	6 (30)	5 (17.8)	6 (22.2)	13 (37.1)	0.355
Mech ventilation, *n* (%)	3 (15)	4 (14.3)	6 (22.2)	10 (28.6)	0.527
Non-invasive ventilation, *n* (%)	1 (5)	2 (7.1)	4 (14.8)	5 (14.3)	0.672
Deaths, *n* (%)	1 (5)	1 (3.6)	3 (11.11)	2 (5.7)	0.764

yo: years old; PIMS: Paediatric Inflammatory Multisystem Syndrome; sd: standard deviation.

**Table 2:** Components of medical cost by clinical area and by age groups.

Emergency room costs	<1 yo (*n* = 20)	1 to < 5 yo (*n* = 28)	5 to < 10 yo (*n* = 27)	10+ yo (*n* = 35)	*p* value
LOS	$1021.63 ($800.83)	$948.5 ($905.36)	$1594.64 ($4003.14)	$711.76 ($452.64)	0.443
Antimicrobials	$1.47 ($3.14)	$5.93 ($10.48)	$8.32 ($14.58)	$27.78 ($83.13)	0.255
General medication	$0.11 ($0.23)	$0.59 ($1.62)	$1.62 ($3.93)	$2.04 ($5.98)	0.108
Imaging tests	$14.44 ($39.54)	$15.61 ($32.04)	$9.84 ($6.72)	$9.08 ($9.32)	0.477
Lab tests	$94.09 ($33)	$101.84 ($41.2)	$118.94 ($50.37)	$97.12 ($43.63)	0.210
Clinical procedures	$50.82 ($69)	$49.99 ($52.2)	$63.22 ($72.27)	$49.07 ($70.75)	0.887
Hospital ward costs					
LOS	$1129.47 ($846.45)	$1285.86 ($1320.89)	$1479.58 ($2593.26)	$900.26 ($467.51)	0.838
Antimicrobials	$26.26 ($68.85)	$97.44 ($174.74)	$225.03 ($425.65)	$253.52 ($948.92)	0.088
General medication	$2.65 ($10.04)	$2742.37 ($8675.2)	$1641.2 ($8317.95)	$471.95 ($2706.64)	0.054
Imaging tests	$0 ($0)	$33.98 ($86.44)	$33.01 ($82.98)	$7.61 ($32.94)	0.116
Lab tests	$66.46 ($39.74)	$169.3 ($306.02)	$169.28 ($234.58)	$125.73 ($122.99)	0.224
Clinical procedures	$40.92 ($70.65)	$115.76 ($216.75)	$76.92 ($125.1)	$81.07 ($298.14)	0.117
PICU/NICU costs					
LOS	$1560.72 ($1116.64)	$1483.47 ($1416.26)	$2504.29 ($6229.35)	$1128.83 ($705.53)	0.410
Antimicrobials	$5.55 ($15.51)	$15.47 ($38.3)	$64.4 ($296.97)	$46.22 ($142.63)	0.534
General medication	$8.55 ($21.74)	$230.89 ($1193.83)	$95.31 ($313.99)	$484.39 ($2770.32)	0.810
Imaging tests	$2.6 ($11.63)	$19.05 ($80.36)	$2.68 ($5.1)	$14.21 ($71.68)	0.531
Lab tests	$99.27 ($148.07)	$116.27 ($194.65)	$127.79 ($265.47)	$143.22 ($247.74)	0.982
Clinical procedures	$147.85 ($286.83)	$142.21 ($261.23)	$181.07 ($393.4)	$132.17 ($305.89)	0.838

Data shown are mean cost USD (standard deviation); LOS: length of stay; yo: years old.

The results of the AIC and BIC are outlined in Table 3 and the results of the log-gamma model are in Table 4 where four regressors had a positive impact on total cost and two of them had negative effects. Boys were more costly than girls while for each one-year-old increment, the expected total cost was reduced, which was not initially obvious when analyzing the age as a discrete variable. Receiving care at the emergency room may lead to cost savings compared with being hospitalized in other clinical areas for COVID-19, although the estimated effect was significant at a 10% alpha level, (90% confidence intervals are shown). Having a positive bacteria culture test resulted in a direct effect on total cost although it did so at a 10% alpha level, but it may reflect the impact of bacterial coinfection suspected. Comorbidities appeared as a cost driver in patients with COVID-19, particularly for patients admitted to tertiary hospitals.

**Table 3:** Results of the Akaike and Bayesian information criteria.

GLM	*n*	AIC	BIC
Log gamma	108	2095.05	2097.73
Square root gamma	108	2095.05	2097.73
Log gaussian	108	2275.44	2278.12
Square root gaussian	108	2275.44	2278.12
Log poisson	108	670567.10	670569.80
Square root poisson	108	670567.10	670569.80

**Table 4:** Results of the GLM to assess factors associated with the total cost of COVID-19 in children.

Predictors	Marginal/discrete effects	*p value*	(90%) confidence intervals	
Male	$1,663.99	0.044	$302.99	$3,025.00
Age	-$142.88	0.032	-$252.51	-$33.26
Emergency room staying	-$6,139.62	0.074	-$11,801.62	-$477.62
Positive bacteria culture test	$1,319.90	0.075	$99.37	$2,540.42
Non-invasive ventilation	$7,678.71	0.138	-$844.47	$16,201.88
Comorbidities	$1,596.67	0.043	$301.27	$2,892.08

## Discussion

According to our results, the average cost per child with COVID-19 in Mexico was $5,943 (95% CI: $4,249–7,637) with the group 5–10 years old showing a maximum cost of US$14,000. Statistically significant regressors were sex, age, staying at an emergency room, having a positive bacterial culture, and having comorbidities. Our cost results were somewhat similar to those of previous studies in the USA (9–12) but specifically for the cases of COVID-19 classified as low severity level. However, our maximum costs estimated differed from those reported in the USA studies for cases identified as having a high severity level. This could be due to high-cost therapies like monoclonal antibodies for COVID-19 in high-risk pediatric patients in some USA hospitals (23), which never happened in the sample hospital. A comparison of our results with those of a study in Korea (13) makes more sense, as both are reported by age groups, with two issues standing out: the first, is the lower costs estimated for all groups, and the second is that they found a positive correlation between age and cost while we evidenced an inverse correlation.

Despite these insights, comparisons between cost studies should be undertaken carefully since there are considerable differences between national healthcare systems, mainly because of initial poor knowledge about the evolution of COVID-19, which possibly gave rise to great heterogeneity regarding medical decision-making and the consumption of hospital resources, and facing patients with chronic conditions and comorbidities, as evidenced in our regression analysis. The low cost estimated in the study for the ER was an expected result derived from the hospital policy that prevents stays longer than 48 h at the ER, whereas the negative effect of staying at the ER on the total cost may be explained by the ability of emergency doctors to stabilize patients. No previous study had reported sex differentials in cost for pediatric patients with COVID-19 as we found for boys being more costly than girls.

The economic implications of COVID-19 remain uncertain in terms of the future, with different effects expected on labor markets, production supply chains, financial markets, and GDP levels (24). Some scholars have argued that the pandemic has increased inter-and intra-country inequalities and has reversed trends in poverty reduction while governmental responses have undermined the multilateral institutions that have thus far facilitated globalization, resulting in growing global uncertainty and higher costs in international transactions (25). Previous studies did not expect a long-term pandemic and posed optimistic assumptions about the pandemic’s decline, making it difficult to portray the pandemic’s current prolonged status and difficult to devise response measures for the COVID-19 economic crisis (4).

Vaccines have proven to reduce the transmission of COVID-19, which can affect economic activity both directly, as consumers and workers feel safer, and indirectly, by allowing governments to relax restrictions hampering economic activity. The effect of higher vaccination rates on economic variables has been evaluated at the county level in the USA, revealing that in response to a 1% point increase in initiated vaccination rates spending rises by 1.3 percentage points (26). The economic evaluation of COVID-19 vaccination in Mexico will require information about the cost of the disease either in adult or pediatric populations.

Cost drivers analyses are relevant as the omicron wave has shown that serious economic disruptions can occur even in highly vaccinated populations (27). This has motivated recommendations by The Lancet Commission on lessons for the future from the COVID-19 pandemic, especially regarding the strengthening of national health systems, increasing investments in primary and public health, investments in effective surveillance systems, adequately trained and resourced outbreak investigation, and response capacity and communications expertise (27). The future may require economically evaluating some antiviral treatments that were given emergency authorization for use for children with COVID-19 (28, 29), so the Mexican health authorities will need cost-effectiveness evidence to aid reimbursement decisions and this study may help.

This study has some limitations. Firstly, the clinical records data were not intended for economic data collection, which could limit the recognition of medical resources consumed during healthcare delivery. Secondly, the hospital accounting system made the identification of unit costs for some resources difficult, meaning some plausible assumptions were necessary for cost estimations. Thirdly, the sample size used could also imply a limitation, since the statistical significance of some coefficients was evaluated with 90% confidence interval. Finally, generalization of our results at the national level could only be possible towards tertiary pediatric hospitals in Mexico, a limitation that arises from the country’s segmented healthcare system. Despite these limitations, these cost estimates still contribute to potential model-based cost-effectiveness studies on future treatments for COVID-19 in children available in Mexico.

## Data availability statement

The data analyzed in this study is subject to the following licenses/restrictions: The data were extracted from medical records of the Hospital Infantil de México Federico Gómez. Requests to access these datasets should be directed to JG-E, juan.gardunoe@gmail.com.

## Ethics statement

Ethical review and approval was not required for the study on human participants in accordance with the local legislation and institutional requirements. Written informed consent from the participants was not required to participate in this study in accordance with the national legislation and the institutional requirements.

## Author contributions

AR-L: responsible for data analysis and writing of methods, results and discussion. RJ-J: responsible for data collection and data base preparation. GS-E: responsible for cost estimation of health personnel. MA-R: responsible for literature review and writing of introduction. SM-V: contribution in literature review and writing of introduction. VG-G: contribution in editing final manuscript. JG-E: responsible for general coordination of tasks. All authors contributed to the article and approved the submitted version.

## Conflict of interest

The authors declare that the research was conducted in the absence of any commercial or financial relationships that could be construed as a potential conflict of interest.

The handling editor GM is currently organizing a Research Topic with the author GS-E.

The handling editor GM declared a past collaboration [10.1007/S12098-021-03959-3 (2021) and 10.1016/J.ARCMED.2022.04.003 (2022) with the author(s) JG-E.

## Publisher’s note

All claims expressed in this article are solely those of the authors and do not necessarily represent those of their affiliated organizations, or those of the publisher, the editors and the reviewers. Any product that may be evaluated in this article, or claim that may be made by its manufacturer, is not guaranteed or endorsed by the publisher.
